# Papillary thyroid carcinoma with invasion of the trachea

**DOI:** 10.1097/MS9.0000000000002233

**Published:** 2024-06-04

**Authors:** Mohammad Eid Al Mohtasib, Khulood “Mohammad Marwan” Sharabate, Raghad Faisal Yahia Dweik, Fatimah Iyad Azmi Shawar, Daleen Ashraf Azmi Shehadeh

**Affiliations:** a Al Ahli Hospital; b Palestine Polytechnic University, Hebron, Palestine

**Keywords:** bronchoscopic tumour removal, papillary thyroid carcinoma, tracheal invasion

## Abstract

**Introduction:**

Papillary thyroid carcinoma typically has a favourable survival rate and a low recurrence rate. However, extrathyroidal extension has a significant negative impact on survival. Among the extrathyroidal extensions, invasion of the trachea by papillary thyroid carcinoma is rare and serves as a marker of more aggressive tumour behaviour. This case report aims to highlight the unusual clinical course of papillary thyroid carcinoma.

**Case presentation:**

A 75-year-old female patient from Gaza has been diagnosed with papillary thyroid carcinoma in 2020. With mediastinal lymph node invasion. she underwent total thyroidectomy and neck dissection and mediastinal lymph node dissection. After the surgery, the patient did not follow up regularly or receive radioiodine treatment. On 2023 presented with hemoptysis, shortness of breath computed tomography (CT) and bronchoscopy reveal thyroid cancer with tracheal invasion, which has invaded the trachea to the left side. The authors treat the patient by bronchoscopy debulking and sent for oncological management.

**Discussion:**

Papillary thyroid carcinoma is the most common type of thyroid cancer and generally has a good prognosis. Tumour staging through various imaging techniques is crucial for determining the next steps. Cases involving tracheal invasion should undergo bronchoscopy for tumour debulking. Surgical management followed by iodine therapy has shown positive outcomes.

**Conclusion:**

When patients with Papillary thyroid carcinoma have haemoptysis, and the imaging examinations reveal a space-occupying lesion in airway, clinicians should focus on Papillary thyroid carcinoma with tracheal invasion, a bronchoscopic examination must be immediately performed because the subsequent surgical management depends on the degree of tracheal invasion.

## Introduction

Highlights

- Papillary thyroid carcinoma typically has a favourable survival rate and a low recurrence rate.
- Invasion of the trachea by papillary thyroid carcinoma is rare and serves as a marker of more aggressive tumour behaviour.
- It is critical to diagnose papillary thyroid carcinoma with tracheal invasion as early as possible.

Most thyroid carcinomas are of the papillary type. Papillary thyroid carcinoma typically exhibits an indolent behaviour and has a favourable prognosis^1^. However, large invasive tumours and/or distant metastases result in a 40% 5-year survival rate^2^. Thyroid carcinoma most commonly metastasizes to the lung, bone, and mediastinal lymph nodes. Cancer invading other structures leads to significant morbidity and mortality. Tumour invasion into the trachea, extending as far as the tracheal lumen, is considered rare, with only a few reported cases^3^. Tracheal invasion can lead to life-threatening symptoms, including difficulty breathing, asphyxia, difficulty swallowing, and bleeding^4^. Here, we present a case of recurrent papillary thyroid carcinoma with tracheal invasion in a 75-year-old woman who presented with shortness of breath.

## Case presentation

A 75-year-old female patient from Gaza was admitted to our hospital in Hebron, Palestine, in February 2020. The patient was diagnosed with diabetes mellitus and hypertension five years ago, and has a past surgical history of hysterectomy 15 years ago due to benign fibroids. She had no history of smoking. During the admission date, she was in her usual state of health until 2 months ago when she incidentally noticed a slowly progressive mass in her neck, accompanied by dull neck pain that did not radiate. She denied experiencing any symptoms of dysphagia, breathing difficulties, hemoptysis, cough, nervousness, weight loss, palpitations, insomnia, hypersomnia, sweating, tremors, nausea, vomiting, changes in bowel habits, frequent urination, or fatigue. Upon neck examination, a well-defined, midline to right side, multinodular 4 ×3 cm solid mass is palpated. The mass was found to be mobile upon swallowing and not attached to the skin. On the right lateral surface of the neck, a smooth, firm mass was observed, causing deviation of the trachea to the left.

The patient sought medical advice in Gaza and underwent an ultrasonogram on 23/11/2019, which revealed thyroid enlargement with multiple well-defined hypoechoic nodules and significant calcification in the right lobe. The right deep jugular and right superficial lymph nodes are enlarged by 2 cm.

A fine needle biopsy was performed on 24/11/2019, and the pathology report indicated the presence of papillary carcinoma in the right cervical lymph node, indicating metastatic papillary carcinoma.

On 12 January 2019, she underwent a computed tomography scan for her neck, chest, and brain, which revealed the following: a large thyroid mass measuring 5.2×2.9 cm, dense focal calcification, heterogeneous texture, infiltration into the anterior neck area reaching the subcutaneous part, and posterior extension into the prevertebral area causing moderate compression and left deviation of the trachea. The scan also revealed multiple mediastinal nodules, primarily affecting the middle mediastinum, as well as a small 5 mm nodule in the posterior right upper lung.

A PET computed tomography scan was performed, revealing thyroid cancer with metastasis to the bilateral cervical lymph nodes and mediastinal lymph nodes.

On 15 February 2020, the patient underwent a total thyroidectomy with modified radical neck dissection involving bilateral group 2/3/4 and mediastinal lymph node dissection in group 7 by sternotomy.

After the surgery, the patient was sent to the intensive care unit for observation. On the second day post-operation, she was transferred to the surgical department, and then the chest tube was removed. On the 3rd day post-surgery, she developed hypocalcaemia; therefore, she was given calcium and vitamin D.

On 25 February 2020, she was discharged with prescriptions for calcium and vitamin D.

For follow-up by an oncologist and endocrinologist, and possible radioiodine therapy.

Due to the onset of the COVID-19 pandemic and the restrictions on patient transport between Gaza and our hospital in Hebron, she was unable to follow up regularly and did not receive radioiodine treatment.

**Figure 1:**
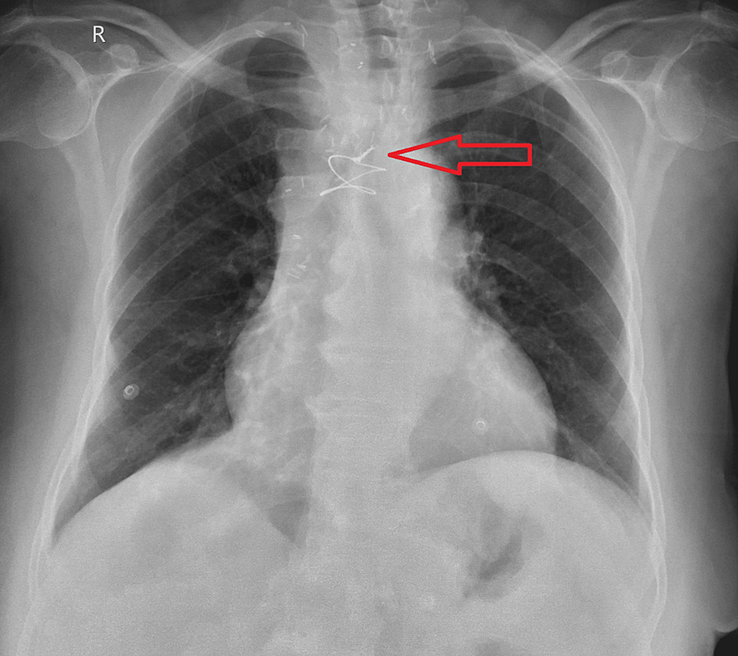
A chest X-ray conducted at the time of admission revealed tracheal deviation to the left side.

On 2 December 2023, the patient presented to our hospital due to sudden onset haemoptysis, dyspnoea that started 4 days before admission. It was associated with heavy chest pain and stridor. Her physical condition further worsened over the course of four days before her admission. A chest X-ray conducted at the time of admission revealed tracheal deviation to the left side. [Figure 1].

**Figure 2:**
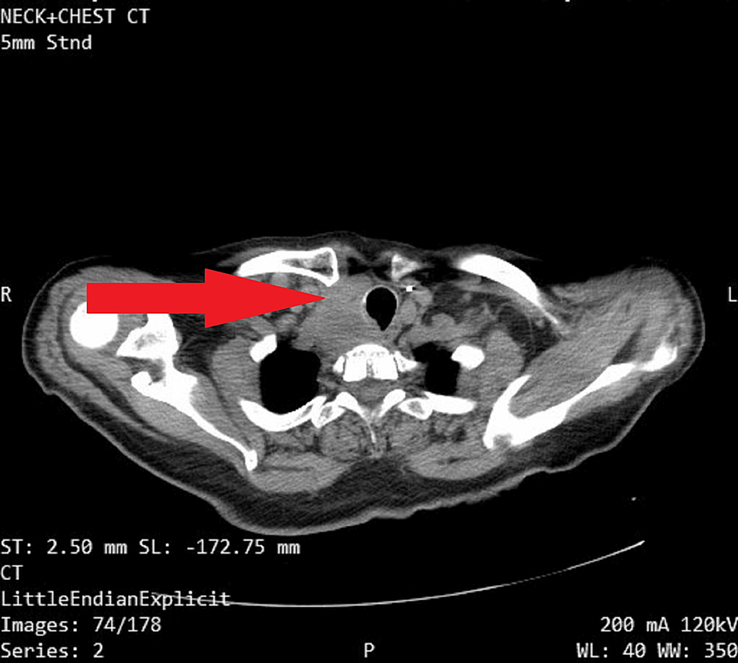
Chest computed tomography (CT) revealed multiple intraparenchymal solid masses in the lower lobe of the right lung, indicating metastasis.

**Figure 3:**
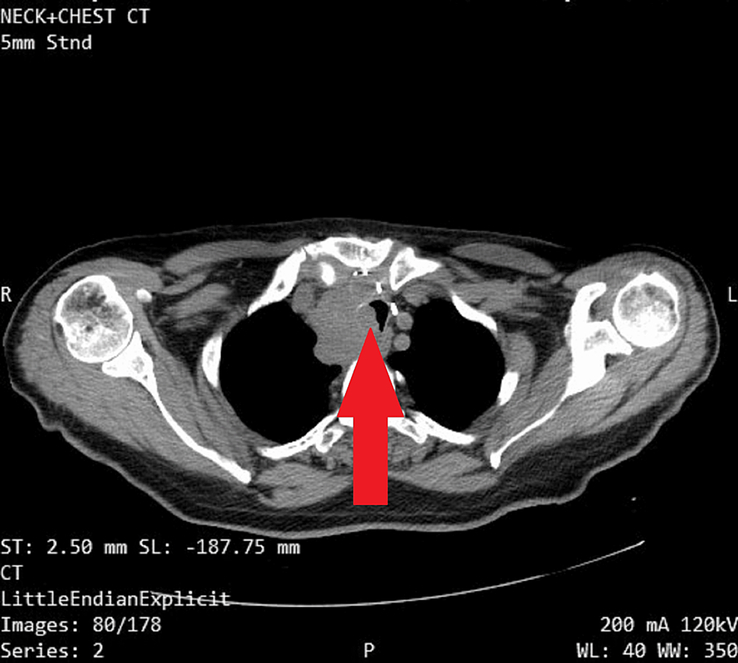
Chest computed tomography (CT) revealed multiple intraparenchymal solid masses in the lower lobe of the right lung, indicating metastasis.

Then we proceeded to a computed tomography (CT) scan of the neck and chest with IV contrast, which revealed a well-defined, heterogeneous, enhancing anterior mediastinal mass measuring ~2.7×4.5×9 cm (anteroposterior diameter×transverse diameter×vertical diameter). The mass invaded and displaced the trachea to the left side, causing severe luminal narrowing. Computed tomography also revealed multiple enlarged mediastinal and cervical lymph nodes with malignant characteristics, most of which exhibited areas of necrosis. The largest node was located in the lower paracervical region, measuring about 2.2×1.7 cm. Furthermore, the chest computed tomography revealed multiple intraparenchymal solid masses in the lower lobe of the right lung, indicating metastasis. The largest mass measured about 9×8 mm. [Figures 2 and 3].

**Figure 4:**
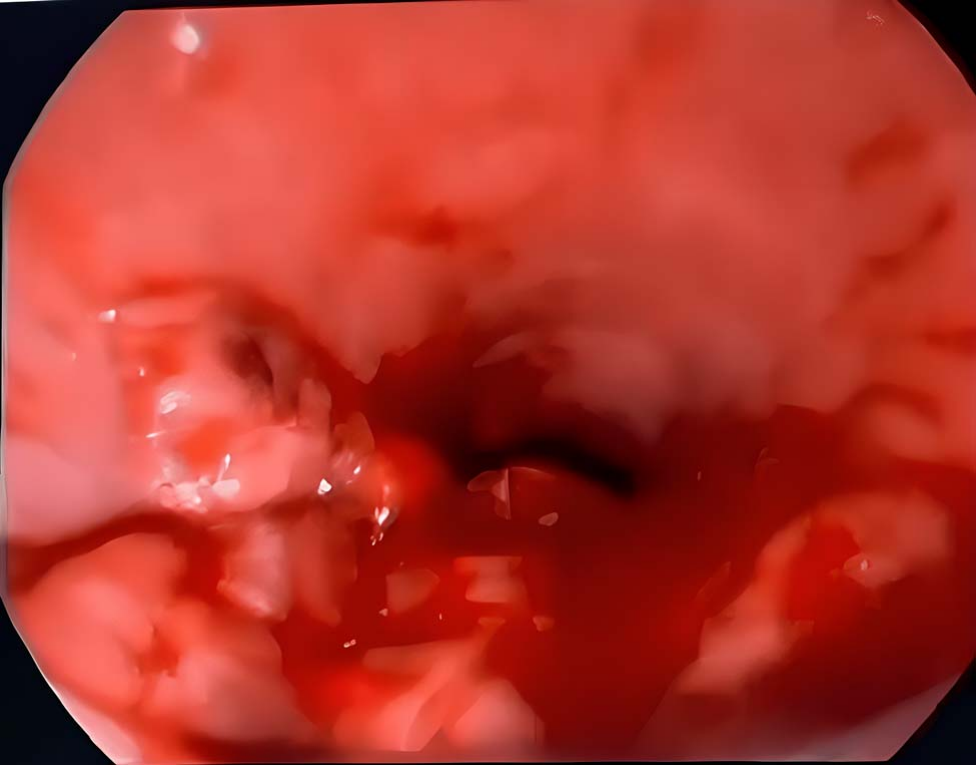
Bronchoscopy shows: endobronchial tumour invasion of trachea.

**Figure 5:**
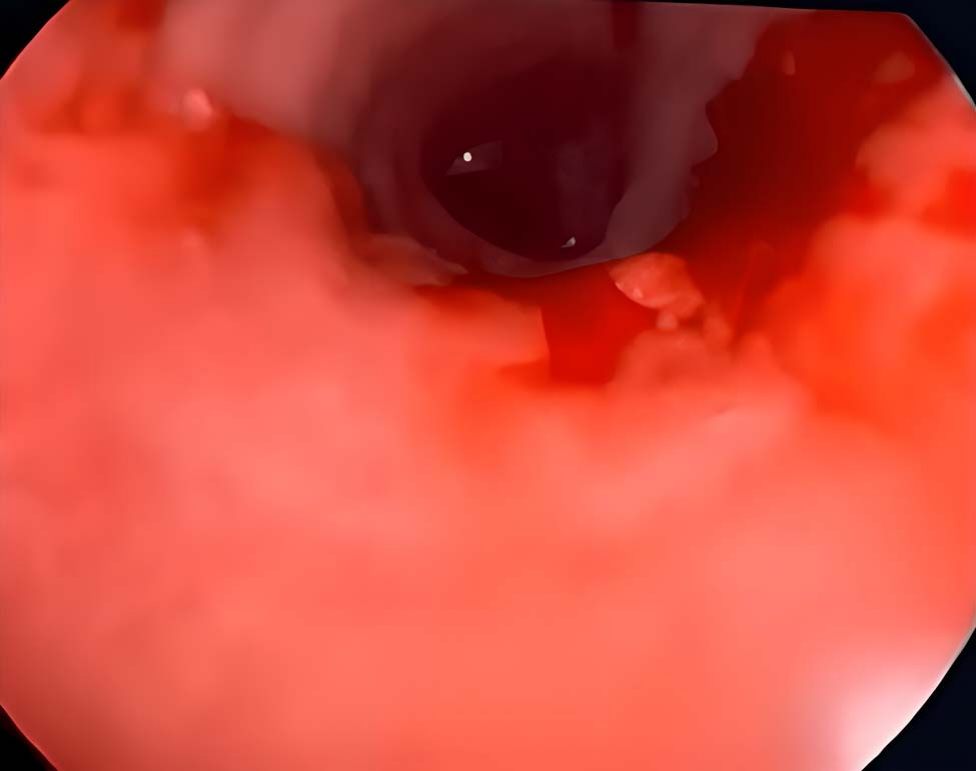
Bronchoscopy shows: endobronchial tumour debulking.

Therefore, due to the patient’s progressive shortness of breath and the imaging findings mentioned above, the patient underwent bronchoscopy and endotracheal debulking to prevent asphyxia, which could occur at any time due to severe tracheal stenosis. [Figures 4 and 5].

The initial bronchoscopy on 15-2-2023 revealed a tracheal mass lesion causing nearly complete obstruction of the lumen. Enucleation and resection were performed using a cold snare via flexible bronchoscopy, and the resected tissue was sent for histopathological examination.

The operation proceeded smoothly, and the patient was transferred to the thoracic surgery ward post-operation. The patient was stable and afebrile. Histopathology revealed papillary thyroid cancer.

Histopathology result:

**Table:** 

Tumour focality	Multifocal (on the same side)
Tumour laterality:	Right
Tumour size:	5×4×3 cm
Histologic type:	Papillary thyroid carcinoma
Histologic grade:	Well to moderately differentiated
Margins	Involved by tumour
Tumour capsular invasion:	Present
Lymphovascular invasion:	Present, extensive, involving more than four vessels
Perineural invasion.	Was not identified
Extrathyroidal extension:	Present (minimal)
Extent of invasion:	T2 (tumour larger than 4 cm, confined to the thyroid with minimal spread)) Extra-thyroid extension)

**Figure 6:**
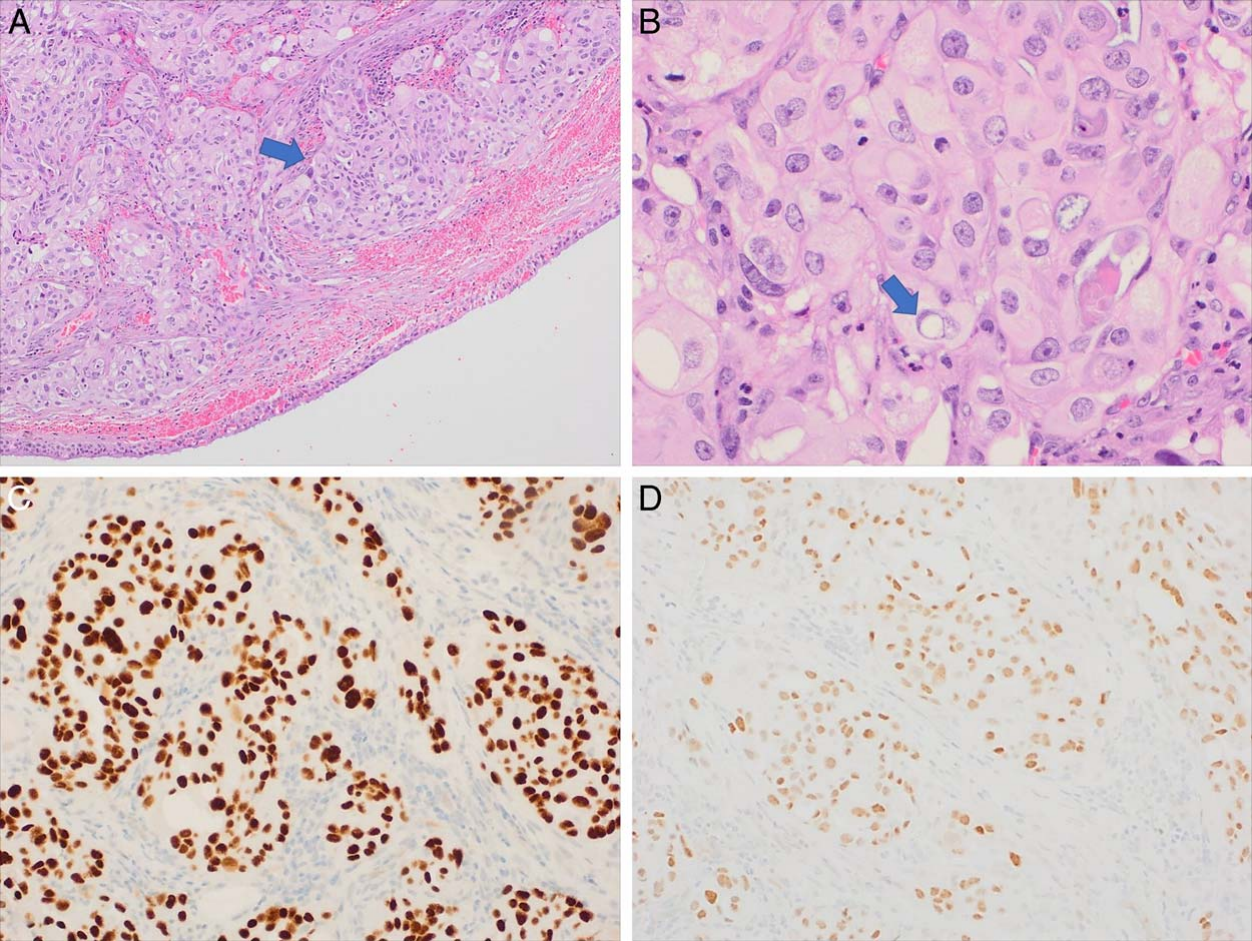
Metastatic carcinoma of thyroid gland origin. (A) Section shows presence of tracheal submucosal tumour predominantly composed of oncocytic cells (arrow) (hematoxylin and eosin, 10×); (B) The tumour cells show abundant eosinophilic cytoplasm, frequent prominent nucleoli, and occasional intranuclear cytoplasmic pseudoinclusions (arrow) (40×); (C) The tumour cells are positive for TTF1 and (D) Pax8 immunostains confirming thyroid origin (20×). The tumour cells are negative for P40 (a squamous cell marker) (not shown).

After that, the patient underwent a second bronchoscopy on 25-2-2023, which revealed a patent trachea Microscopic picture of metastatic carcinoma of thyroid gland origin Figure 6.

## Discussion

papillary thyroid carcinoma is the dominant form of thyroid cancer that accounting for 80–85% of all thyroid cancer cases, it is well known for its favourable prognosis and survival rate^5^. the incidence of invasion by Papillary thyroid carcinoma is extremely low^6^. The extrathyroidal extension (in which the tumour invades the adjacent structures including striated muscles, trachea, larynx, jugular vein, carotid artery, oesophagus, and recurrent laryngeal nerve) is universally considered a conspicuously adverse prognostic factor^7^, and its incidence in Papillary thyroid carcinoma is about 5–34%^7^. according to The International Union Against Cancer TNM classification system of primary thyroid tumour, this case classifies as (T4a, N1b, M1), this classification is the most widely-used staging system for extrathyroidal extension worldwide.

Papillary thyroid carcinoma with tracheal invasion can extend into the tracheal lumen, with an incidence of 0.5–1.5%^8^, constituting 35–60% of all cases with invasion.

According to Shin’s staging of tracheal invasion, this case is classified as stage IV^9^.

Papillary thyroid carcinoma with tracheal invasion can extend into the tracheal lumen, with an incidence of 0.5%-1.5%^8^, constituting 35–60% of all cases involving invasion. According to Shin’s staging in, this case is classified as stage IV of tracheal invasion^9^.

Tumours with tracheal invasion can lead to airway obstruction, which is the primary cause of death in cases of thyroid tumours. Preoperative clinical signs of tracheal invasion include hemoptysis, hoarseness, the presence of a cervical mass^10^, and shortness of breath, as in this case.

Imaging examinations, including ultrasound, computed tomography (CT), and MRI, are commonly performed in cases of thyroid tumours with tracheal invasion. Ultrasound has a unique advantage over other imaging examinations in showing the extension of primary thyroid tumours and diagnosing the preoperative staging of thyroid tumours with lymph node metastasis, with an accuracy reaching 83.3%^11,12^. Therefore, ultrasound is the preferred method for diagnosing thyroid cancer with tracheal invasion, even in the early stages.

The bronchoscopy examination is typically conducted in patients suspected of having thyroid tumours with tracheal invasion to assess the extent of laryngotracheal invasion and enable direct observation of changes in the bronchial mucosa^13^. It is also used to remove a portion of the tumour, particularly when it causes significant obstruction in the tracheal lumen, resulting in shortness of breath. In one instance, bronchoscopy was performed twice to remove the majority of the endotracheal tumour. Surgical management is a good choice for cases of papillary thyroid carcinoma. Thyroidectomy is necessary, along with radical resection for invaded lymph nodes. In cases of tracheal invasion, shave excision of the affected tissue or full-thickness tracheal resection can be performed^14^. In this particular case, after total thyroidectomy with radical resection of cervical and mediastinal lymph nodes, the tumour invaded the trachea and lungs. Chemotherapy and radioiodine I131 are preferred for use after debulking the tumour by bronchoscopy.

Although papillary thyroid carcinoma demonstrates a satisfactory prognosis, 8–28% of patients with this condition experience tumour recurrence. There are multiple risk factors for recurrent or persistent tumours in this case, including being female, age over 45, invasion of both lobes of the thyroid gland (bilaterally), extension beyond the thyroid capsule, and invasion of lymph nodes. Another important factor contributing to recurrent papillary thyroid carcinoma in this case is the patient’s failure to follow up or undergo iodine ablation therapy (Iodine131) for 30 months after thyroidectomy. In other words, she did not complete her therapy. This could be attributed to the fact that postoperative I131 treatment can reduce the risk of recurrence and improve the survival rate.

## Conclusion

Overall, the postoperative survival rate of patients with PTC is generally excellent. If patients have the clinical manifestations of tracheal invasion, they should immediately undergo imaging examinations such as US, CT, or MRI.

When patients with Papillary thyroid carcinoma have haemoptysis, and the imaging examinations reveal a space-occupying lesion in the thyroid and airway, clinicians should focus on Papillary thyroid carcinoma with tracheal invasion, a bronchoscopic examination must be immediately performed because the subsequent surgical management depends on the degree of tracheal invasion.

## Ethical approval

The study is exempt from ethical approval in our institution.

## Consent

Written informed consent was obtained from the patient for publication of this case report and accompanying images. A copy of the written consent is available for review by the Editor-in-Chief of this journal on request.

## Source of funding

The study did not receive any financial help.

## Author contribution

Data collection: M.E.A.M. Study concept or design: M.E.A.M. Writing the manuscript: M.E.A.M., R.F.y.D., F.I.A.S., K.“mohammad marwan”S., D.A.A.S. Review and editing the manuscript: M.E.A.M.

## Conflicts of interest disclosure

The authors have no conflicts of interests to declare.

## Research registration unique identifying number (UIN)

The study does not have a trial registry number.

## Guarantor

Mohammad Al Mohtasib.

## Data availability statement

Not applicable.

## Provenance and peer review

Not commissioned, externally peer-reviewed.
